# Uric Acid and Chronic Kidney Disease

**DOI:** 10.1016/j.xkme.2026.101411

**Published:** 2026-05-14

**Authors:** Srinath Yadlapalli, Divya K. Nekkalapudi, Jay B. Wish

**Affiliations:** 1 Division of Nephrology, Indiana University School of Medicine, Indianapolis, IN; 2 Division of Rheumatology, Indiana University School of Medicine, Indianapolis, IN

**Keywords:** Chronic kidney disease (CKD), uric acid, hyperuricemia, urate-lowering drugs, xanthine oxidase inhibitors

## Abstract

The prevalence of hyperuricemia is high among people with chronic kidney disease (CKD), increasing significantly as kidney disease progresses. Whether this is an association or a cause-and-effect relationship remains controversial. While evidence strongly supports the treatment of symptomatic hyperuricemia in people with CKD, the evidence supporting the treatment of asymptomatic hyperuricemia is mixed. Some studies show that treatment retards CKD progression, while others do not. In this review, we discussed uric acid metabolism, its association with CKD, and the use of uric acid-lowering agents. We also examined studies conducted to determine whether treating hyperuricemia is beneficial. We hope to provide nephrologists with the knowledge and confidence to confront hyperuricemia, specifically addressing management of asymptomatic hyperuricemia, the differences among society guidelines worldwide, which urate-lowering drugs should be chosen, the role of non-pharmacological therapy in patients with CKD, and the need to minimize the use of medications that may increase uric acid levels.

## Introduction

The association between serum uric acid (SUA) level and kidney disease has been known for almost 2 centuries. In 1859, reports described white deposits, composed of urate, in the kidneys of numerous patients with gout.^1^^,^^2^ At that time, except for the treatment of acute gout episodes with colchicine, few treatment options were available. However, this changed with the discovery of the uricosuric drug probenecid in the 1950s and the xanthine oxidase inhibitor allopurinol in the 1960s.^3^ More recently, the approval of agents including febuxostat by the US Food and Drug Administration (US FDA) in 2009 and pegloticase in 2010 has been game-changing.^4^

Hyperuricemia, defined as SUA level > 6 mg/dL in women and > 7 mg/dL in men, affects 38 million Americans, and its incidence increases with the adoption of the Western diet.^5^ In one study, the prevalence of SUA levels > 7 mg/dL in US adults was 11.9% overall, with 20.2% in men and 4.2% in women in 2015-2016.^6^ Women tend to have lower uric acid levels, likely due to increased uric acid excretion in response to estrogen.^7^ Hyperuricemia is frequently observed in pre-eclampsia and may serve as a predictor for its severity.^8^ Hyperuricemia is a risk factor for hypertension,^9^^,^^10^ diabetes mellitus,^11^ nephrolithiasis, and gout. Additionally, it is associated with cardiovascular disease (CVD),^12^ metabolic syndrome,^13^ and cognitive decline.^14^^,^^15^

The prevalence of chronic kidney disease (CKD) is estimated to be around 11% in the US and is primarily caused by hypertension and diabetes.^16^ Hyperuricemia is a risk factor for both hypertension and diabetes. In addition, CKD is a known risk factor for CVD,^17^ and vice versa, with both being associated with hyperuricemia. Uric acid is a major extracellular antioxidant; however, it acts as an intracellular pro-oxidant.^18^^,^^19^ The relationship between CKD and SUA level has shown a U-shaped pattern in some studies,^20^ but other studies have shown a J-shaped relationship.^21^ This complex interplay of risk factors leads to a crucial question: does reduction of uric acid halt the progression of CKD?

## Uric Acid Metabolism

### Uric acid synthesis

**Figure 1:**
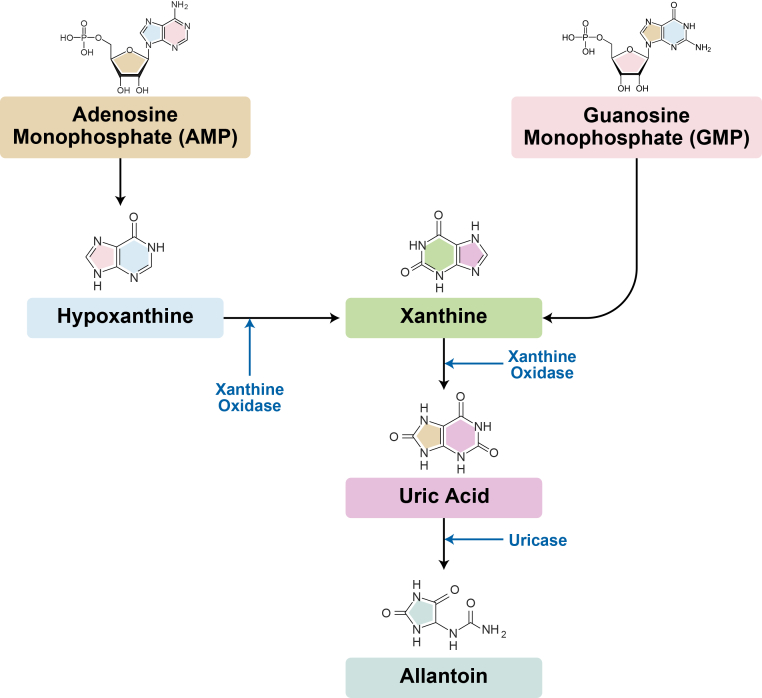
Key steps in purine metabolism.^18^^,^^19^ AMP, adenosine monophosphate; GMP, guanosine monophosphate

Uric acid is the end product of purine metabolism, with key steps shown in Fig 1.^18^^,^^19^ Purines, including adenine and guanine, originate from both exogenous and endogenous sources. Dietary intake accounts for 100-200 mg/d of purines, while endogenous production from dead cells contributes 500-600 mg/d. Key endogenous sites of purine generation include the liver, intestines, muscles, kidneys, and vascular endothelium.^13^^,^^22^

### Uric acid and diet

**Figure 2:**
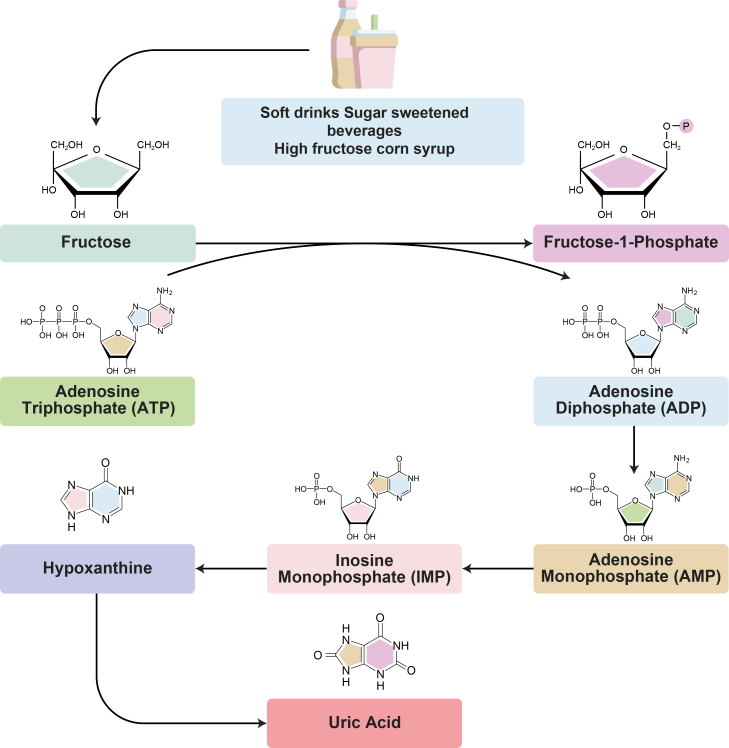
Metabolism of fructose to uric acid. ADP, adenosine diphosphate; AMP, adenosine monophosphate; ATP, adenosine triphosphate; IMP, inosine monophosphate.

In recent decades, a progressive rise in SUA levels has been observed, particularly in populations adhering to Western diets. This trend has spurred interest in the role of specific dietary approaches, such as the Dietary Approaches to Stop Hypertension diet,^23^ the Mediterranean diet, plant-based diet^24^ and low-purine diets,^25^ in managing hyperuricemia and gout. A 2018 meta-analysis conducted by Major and Topless^26^ identified seven foods associated with elevated uric acid levels: beer, liquor, wine, potatoes, poultry, soft drinks, and meats (beef, pork, or lamb). Conversely, 8 foods were linked to reduced uric acid levels: eggs, peanuts, cold cereal, skim milk, cheese, brown bread, margarine, and noncitrus fruits. Despite these associations, the authors concluded that, in contrast to genetic contributions, diet explains very little variation in serum urate levels in the general population. This was confirmed by a 2021 study by Topless et al,^27^ which demonstrated that diet has a relatively minor role in determining hyperuricemia compared to inherited genetic variants and body mass index.^27^ In contrast, other studies suggest that the increasing rate of hyperuricemia is attributed to the consumption of fructose-containing sugars, including soft drinks and fruit juices. In a systematic review and meta-analysis of prospective cohort studies assessing the relation of food sources containing fructose sugars with incident gout and hyperuricemia involving 154,289 participants, an adverse association of sugar-sweetened beverages and fruit juice intake with incident gout was found, but it did not extend to fruit intake.^28^ Fructose is a major component of added sugars, fruits, and high fructose corn syrup and causes intracellular adenosine triphosphate (ATP) level depletion and production of uric acid.^29^^,^^30^ The metabolism of fructose to uric acid is shown in Fig 2.^31^^,^^32^

### Uric acid excretion

Approximately two-thirds of uric acid excretion is by the kidneys, while the remaining one-third is by the intestine.^13^ In the blood, uric acid primarily exists as urate, which is more soluble. Humans exhibit high levels of serum urate; in contrast, other mammals maintain low serum urate levels because of the presence of the enzyme uricase, which converts urate to the highly soluble and easily excreted allantoin. Humans lack the enzyme uricase.

In the kidneys, uric acid undergoes filtration, reabsorption, and secretion. Because it is not protein-bound, uric acid is easily filtered by the glomerulus; however, 95% of it is reabsorbed by the proximal tubule. Accounting for secretion, the fractional excretion of uric acid is 10%, meaning that 10% of filtered uric acid is ultimately excreted in the urine.

**Figure 3:**
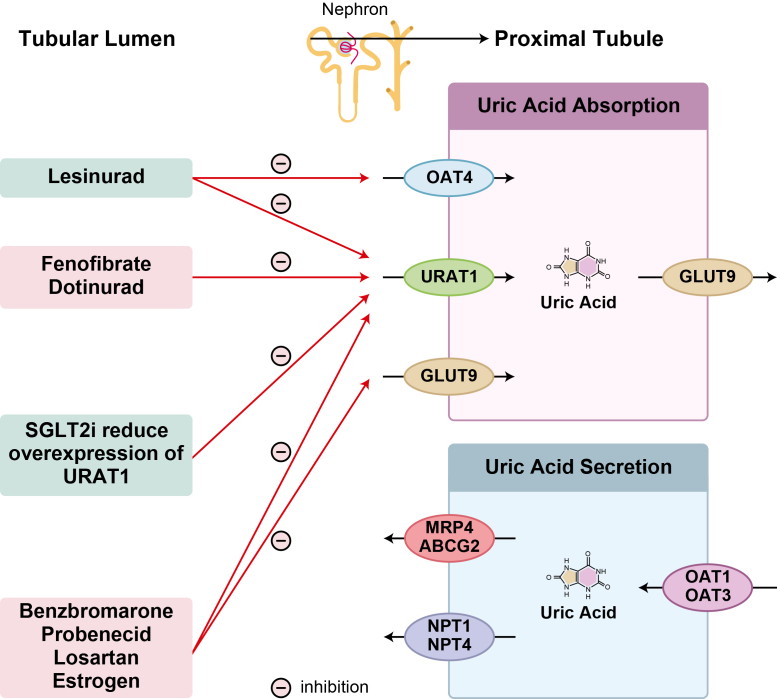
Uric acid absorption and secretion in the proximal tubule of the kidney and the effect of urate-lowering therapies on uric acid transporters. ABCG2, ATP-binding cassette transporter subfamily G protein; GLUT9, glucose transporter 9; MRP4, multidrug resistance protein 4; NPT, sodium-dependent phosphate transporter; OAT, organic anion transporter; SGLT2i, sodium-glucose co-transporter inhibitor; URAT1, urate transporter 1.

Within the proximal renal tubule, urate reabsorption is primarily facilitated by 2 key transporters: urate transporter 1 (URAT1) and glucose transporter 9 (GLUT9), as shown in Fig 3. URAT1, a member of the organic anion transporter (OAT) family, resides in the apical membrane. GLUT9 is situated in the apical and basolateral membranes.

The transporters that mediate urate secretion in the basolateral membrane are OAT1 and OAT3, as shown in Fig 3. In the apical membrane, urate secretion is mediated by sodium-dependent phosphate transporters 1 and 4, the multidrug resistance protein 4 channel, and a member of the ATP-binding cassette transporter subfamily G protein.^13^^,^^18^^,^^22^

Genetic mutations of transporters that mediate reabsorption (URAT1 and GLUT9) lead to hypouricemia. Conversely, genetic mutations of transporters that mediate secretion (sodium-dependent phosphate transporter 1 and 4, and the ATP-binding cassette transporter subfamily) lead to hyperuricemia.^19^

## Hyperuricemia and CKD

### Association

Several observational studies have demonstrated an association between SUA levels and CKD progression. Table 1^33^, ^34^, ^35^, ^36^, ^37^, ^38^, ^39^, ^40^ provides a summary of these publications. Though many studies showed a positive correlation between SUA level and CKD, a few studies, including the Modification of Diet in Renal Disease study, did not.

**Table 1:** Studies of the Association of Hyperuricemia and Kidney Disease

Study	Design	Participants	Results
Hsu et al^33^ (2009)	Large cohort study in California	177,570 patients were included in a 25-year follow-up	Higher SUA was an independent risk factor for ESKD
Wang et al^34^ (2011)	Retrospective cohort study in Taiwan	94,422 patients with a mean of 3.5 years of follow-up	Hyperuricemia is a risk factor for CKD in Taiwan Adjusted HR of 1.03 (95% CI, 1.01-1.06)
Zhu P et al^35^ (2014)	Meta-analysis of cohort studies	15 cohorts, including 99,205 individuals	Positive association between SUA level and risk of CKD independent of established metabolic risk factors RR 1.22 (95% CI, 1.16-1.28) per 1 mg/dL SUA level increment
Ling Li et al^36^ (2014)	Systematic review and meta-analysis	13 studies, including 190,718 participants	High SUA level is an independent predictor for newly diagnosed CKD in non-CKD patients (OR, 2.35 with 95% CI, 1.59-3.46)
Gonclaves et al^37^ (2022)	Systematic review and meta-analysis	18 studies on CKD incidence (n = 398,663) and 6 studies on CKD progression (n = 13,575)	Lower SUA levels were protective for CKD incidence (RR, 0.65 with 95% CI, 0.56-0.75) and progression (RR, 0.55 with 95% CI, 0.44-0.68)
Lee et al (2025)^38^	Data analysis of the AASK study (a randomized 3 × 2 factorial trial)	1094 African Americans with hypertensive kidney disease were enrolled in the AASK study. Investigators analyzed 713 participants with a 12-year follow-up, dividing them into five groups.	When compared with the normo-increasing group, the high stable (HR, 2.28, *P* = .012), the High Increasing (HR, 1.95; *P* = .042), and extremely high stable (HR, 3.21, *P* < .001) uric acid groups had a higher risk of ESKD outcomes, even after adjusting for the *APOL1* risk allele, antihypertensive drug class, and blood pressure targets.
Vlachopanos et al (2026)^39^	Cross-sectional study	A total of 446 treatment-naive, newly diagnosed patients with hypertension were divided into 3 groups based on SUA levels.	Treatment-naive hypertensive patients with elevated SUA levels demonstrated reduced kidney function compared with those with low SUA.
MDRD study^40^	Randomized controlled trial	The study, which included 840 participants, was testing the effect of dietary protein restriction and blood pressure control on kidney disease progression.	In CKD3-4 patients, hyperuricemia was not an independent risk factor for kidney failure.

AASK, African American Study of Kidney Disease and Hypertension; CI, confidence interval; CKD, chronic kidney disease; ESKD, end-stage kidney disease; MDRD, Modification of Diet in Renal Disease; OR, odds ratio; RR, relative risk; SUA, serum uric acid.

### Pathophysiology

The mechanism by which uric acid causes kidney damage is thought to be 2-fold: crystal-dependent and crystal-independent.

#### Crystal-Dependent Mechanism

In the crystal-dependent mechanism, hyperuricosuria promotes the formation of uric acid crystals, particularly in acidic urine. These crystals adhere to the tubular epithelium and cause local inflammation, which can lead to the rupture of the tubular wall. This allows crystals to escape into the interstitium, resulting in further inflammation, reactive oxygen species, and increased oxidative stress.^41^ This explains why, before the availability of urate-lowering therapies (ULT), autopsies of patients with gout universally showed kidney disease, with urate crystals found in the outer medulla in 90% of cases.^2^^,^^42^^,^^43^

The importance of renal urate load is further illustrated by genetic variants. Hypouricemia is uncommon, with a reported prevalence of 0.3% in a Japanese study, and often arises from *SLC22A12* mutations encoding the URAT1 transporter.^44^ These patients are predisposed to exercise-induced acute kidney injury and urinary stones, a mechanism observed in a *Urat1-Uox* (uricase) double-knockout mouse model. Although baseline serum levels are low, anaerobic exercise triggers a temporary rise in SUA levels and urinary excretion, potentially causing intratubular crystallization and injury. Animal and clinical data indicate that this form of AKI can be blocked by xanthine oxidase inhibitors, which limit the transient surge of uric acid into the tubules regardless of baseline levels.^45^^,^^46^ These findings provide a mechanistic explanation for epidemiological J-shaped and U-shaped curves, as individuals with low serum urate levels may carry *URAT1* mutations that paradoxically increase crystal-induced renal injury.

Similarly, the ATP-binding cassette transporter subfamily protein dysfunction highlights the pathogenic role of renal urate load. In addition to its role in the proximal tubule, this transporter secretes uric acid into the intestinal lumen; its defect reduces intestinal excretion, raising SUA levels and triggering compensatory kidney excretion via decreased URAT1 and GLUT9 reabsorption. In affected individuals, risk factors such as low urine pH or heat stress promote urate crystallization.^47^ Notably, low urine pH is an independent predictor of CKD.^48^ In a prospective study of 343 patients with gout, combining citrate-based alkalinization with febuxostat was associated with a lower urinary albumin-to-creatinine ratio and fewer gout flares.^49^

#### Crystal-independent mechanism

Independent of crystals, an elevated level of SUA drives kidney damage through an integrated cascade of metabolic and hemodynamic changes. While uric acid acts as an extracellular antioxidant, elevated SUA levels drive its uptake into cells, where it paradoxically shifts to a pro-oxidant role. This stimulates reactive oxygen species production, inducing oxidative stress and direct tubule cell injury. Furthermore, hyperuricemia causes endothelial dysfunction by impairing nitric oxide production. This environment triggers the renin-angiotensin-aldosterone system, increasing renin, aldosterone, and angiotensin II, which promote renal vasoconstriction, sodium retention, and hypertension. The subsequent development of afferent arteriolopathy leads to a loss of renal autoregulation, resulting in glomerular hypertension. In addition to these processes, high SUA levels induce epithelial-mesenchymal transition, transforming epithelial cells into myofibroblasts. These myofibroblasts drive tubulo-interstitial fibrosis, shifting the pathology from a metabolic disturbance to permanent structural kidney damage.^13^^,^^41^^,^^50^

## ULT

### Xanthine oxidase inhibitors

#### Allopurinol

Allopurinol is a purine analogue that inhibits xanthine oxidase and hence decreases SUA level. It is metabolized to oxypurinol predominantly in the liver. Oxypurinol is then excreted by the kidneys. Allopurinol can cause a range of adverse effects, from a mild maculopapular eruption to severe cutaneous adverse reactions. These severe cutaneous adverse reactions include serious conditions such as Stevens-Johnson syndrome/toxic epidermal necrolysis, drug reaction with eosinophilia and systemic symptoms, and allopurinol hypersensitivity syndrome. Fortunately, the incidence of allopurinol-induced Stevens-Johnson syndrome/toxic epidermal necrolysis is low, reported at 0.69 per 1,000 person-years in a large cohort study. To mitigate the risk of these side effects, allopurinol therapy typically begins at a lower dose and is gradually increased.^51^

The HLA-B*58:01* allele is associated with an increased risk of severe cutaneous adverse reaction to allopurinol. This heightened risk is because of the ability of oxypurinol to induce a T-cell response that is more pronounced in patients carrying the *HLA-B*58:01 allele.

CKD is also a known risk factor for allopurinol hypersensitivity. Given the higher prevalence of the HLA-B*58:01* allele in Asian populations and the added risk associated with CKD, it is generally recommended that *HLA-B*58:01 testing be considered, particularly for Asian patients with CKD, before initiating allopurinol therapy.^52^

#### Febuxostat

Febuxostat is a nonpurine xanthine oxidase inhibitor that is predominantly metabolized in the liver. Concerns for increased cardiovascular mortality observed in the Cardiovascular Safety of Febuxostat or Allopurinol in Patients with Gout trial resulted in a black box warning from the US FDA.^53^ However, this concern was not replicated in subsequent studies.

### Uricosurics

Benzbromarone and probenecid act on the URAT-1 and GLUT-9 in the proximal renal tubule, thereby increasing renal urate excretion. Benzbromarone is associated with liver toxicity and is not available in the US. Probenecid is the only uricosuric drug available in the US.^54^ Lesinurad inhibits URAT-1 and OAT-4. It was linked to excessive acute kidney injury, especially as a monotherapy, and is not available in the US.^55^ Sulfinpyrazone is not available in the US because of its risk to bone marrow function.^56^

### Uricases

#### Pegloticase

Pegloticase is a pegylated, recombinant uricase that degrades uric acid to more soluble allantoin. It was approved by the US FDA in 2010. It has a half-life of 14 days and is administered intravenously every 2 weeks. Common side effects include gout flare and infusion reactions.^57^ The action of pegloticase on uric acid produces hydrogen peroxide. Red blood cells that lack the glucose-6-phosphate dehydrogenase enzyme are susceptible to oxidative damage. Thus, pegloticase is contraindicated in patients with glucose-6-phosphate dehydrogenase deficiency.^58^ Pegloticase is US FDA-approved only for refractory chronic gout and not for tumor lysis syndrome.^57^ However, the use of pegloticase in tumor lysis syndrome has been reported.^59^

#### Rasburicase

Rasburicase is a recombinant urate oxidase that promotes the degradation of uric acid into allantoin. Similar to pegloticase, it is contraindicated in patients with glucose-6-phosphate dehydrogenase deficiency and can cause hemolysis and methemoglobinemia. Another limiting factor of rasburicase is its high cost.^60^ The half-life of rasburicase is short, approximately 21 hours. Due to repeated gout flares and potentially immunogenic reactions, it is not used for chronic gout. It should be noted that rasburicase is nonpegylated and highly immunogenic. The PEGylation of the uricase enzyme with polyethylene glycol, as with pegloticase, decreases the antigenicity and increases the half-life.^61^

### Other drugs

#### Losartan

Losartan is an angiotensin II receptor antagonist that inhibits URAT-1 and GLUT9. It promotes uric acid excretion.^56^ This is unique to losartan and not significantly apparent in other angiotensin receptor blockers. The reason for the higher affinity of losartan to the urate transporters is not known.^62^^,^^63^

#### Fenofibrate

Fenofibrate increases uric acid excretion by inhibiting URAT-1.^13^^,^^56^

#### Sodium glucose cotransporter 2 inhibitors

In a pooled meta-analysis of the Dapagliflozin and Prevention of Adverse Outcomes in Heart Failure trial, it was found that dapagliflozin-treated patients were less likely to initiate ULT.^64^^,^^65^ In a post hoc analysis of the Empagliflozin Outcome Trial in Patients with Chronic Heart Failure and a Reduced Ejection Fraction (EMPEROR-Reduced) trial, the effects of empagliflozin on SUA level and gout were evaluated. Empagliflozin significantly decreased SUA levels within the first 4 weeks, and this reduction remained stable throughout the study.^64^^,^^66^ Sodium glucose cotransporter 2 inhibitors may reduce the overexpression of URAT1 by improving insulin resistance.^13^

Figure 3 illustrates the effect of various drugs on urate transporters.

## Effects of Intervention

Because of its pathophysiology and the association between hyperuricemia and CKD, multiple studies have been conducted to determine if ULT can prevent the progression of CKD.

### Xanthine Oxidase Inhibitors

#### Allopurinol

Table 2^67^, ^68^, ^69^, ^70^, ^71^ summarizes the studies examining the effect of allopurinol on kidney disease progression. Two large randomized controlled trials did not demonstrate a benefit of SUA level reduction with allopurinol for kidney disease progression. However, it is important to note the limitations in this evidence. In the preventing early renal function loss study, which included type 1 diabetic patients, the inclusion criterion was an SUA level of 4.5 mg/dL or higher at screening. However, the baseline mean SUA level was only 6.1 ± 1.5 mg/dL.^67^ This is considerably lower than in earlier positive trials, such as Goicoechea et al,^68^ where the mean SUA level was 7.6 mg/dL. Another factor to consider is the mean duration of diabetes, which was 34.6 years. This prolonged duration might suggest that irreversible kidney damage had already occurred, potentially limiting the effectiveness of any intervention. The results from this study can not be generalized to patients with nondiabetic CKD or type 2 diabetes.

**Table 2:** Studies of the Effect of Allopurinol on Kidney Disease Progression

Study	Design	Drug(s)	Participants	Results
Goicoechea et al^68^ (2010)	Prospective Randomized trial	Allopurinol 100 mg daily vs usual therapy	113 patients with eGFR < 60 mL/min/1.73 m^2^	In the control group, eGFR rate decreased by 3.3 ± 1.2 mL/min/1.73 m^2^, and in the allopurinol group, eGFR increased by 1.3 ± 1.3 mL/min/1.73 m^2^ after 24 mo. The conclusion was that allopurinol slows down the progression of renal disease in patients with CKD.
Goicoechea et al^70^ (2015)	Post hoc analysis of long-term follow-up	Allopurinol vs standard treatment	107 participants were followed for an additional 5 years	Only 9 patients in the allopurinol group had a renal event compared with 24 patients in the control group (HR, 0.32 with 95% CI, 0.15-0.69; *P* = .004]. The conclusion was that allopurinol may slow the rate of progression of kidney disease and reduce CV risk)
Bose et al (2013)^71^	Systematic review	Allopurinol	8 trials with 476 participants	In 3 trials, allopurinol treatment abrogated increases in serum creatinine level from baseline (MD, −0.4 mg/dL with 95% CI, −0.8 to 0 mg/dL). Allopurinol may retard the progression of CKD
PERL study (2020)^67^	Double blind randomized placebo-controlled trial	Allopurinol vs placebo	530 patients with type 1 diabetes, eGFR 40 to 99.9 mL/min/1.73m^2^	No benefit of SUA level reduction on kidney outcomes in type 1 DM and early to moderate diabetic kidney disease
CKD-FIX study (2020)^69^	Double blind randomized placebo-controlled trial, including 31 centers	Allopurinol Vs Placebo	369 patients with CKD stage ¾, no gout, who had a UACR value > 265 or an eGFR rate decrease of 3 mL/min in the last year	Allopurinol did not slow the decline in eGFR compared with placebo

CI, confidence interval; CKD, chronic kidney disease; CV, cardiovascular; DM, diabetes mellitus; eGFR, estimated glomerular filtration rate; HR, hazard ratio; MD, mean difference; PERL, preventing early renal function loss; SUA, serum uric acid.

The controlled trial of slowing of kidney disease progression from the inhibition of xanthine oxidase study included patients with stage 3 and 4 CKD who were at high risk for progression, defined by a urinary albumin-to-creatinine ratio ≥ 265 mg/g or a decrease in estimated glomerular filtration rate (eGFR) of at least 3 mL/min/1.73 m^2^ per year. In total, 50%-59% of the CKD cases stemmed from nondiabetic kidney disease. While the mean SUA level at baseline was 8.2 ± 1.8 mg/dL and treatment resulted in a sustained mean reduction of 35%, the study did not demonstrate a delay in CKD progression. However, several limitations must be considered. The study lacked sufficient power because of incomplete enrollment and a high attrition rate, with 87 patients (nearly 24%) failing to complete the trial. Crucially, the study excluded patients with a history of gout, and a high baseline SUA level was not an inclusion criterion. The trial was not specifically designed to determine whether treating hyperuricemia protects against CKD, but rather to evaluate allopurinol as an intervention for the general CKD population.^69^

#### Febuxostat

A summary of the studies examining the effect of febuxostat on kidney disease progression is shown in Table 3,^72^, ^73^, ^74^, ^75^ the results of which are mixed.

**Table 3:** Studies of the effect of febuxostat on kidney disease progression

Study	Design	Drug(s)	Participants	Results
Zhang et al^72^ (2019)	Prospective cohort study in China	Febuxostat vs Allopurinol	152 patients with CKD stage 2/3 and hyperuricemia	Febuxostat was superior in delaying renal impairment progression compared with allopurinol in patients with CKD with hyperuricemia
FREED study (2019)^73^	Prospective randomized open label blinded endpoint	Febuxostat vs conventional therapy	1,070 patients with hyperuricemia at risk of cerebral, cardiovascular, or renal disease	Febuxostat lowers SUA levels and delays the progression of renal dysfunction. Primary composite event rate, including renal events, was significantly lower in the febuxostat group (HR of 0.75 with 95% CI 0.59-0.95; *P* = .017).
FEATHER study (2018)^74^	Randomized double blinded placebo-controlled study in Japan	Febuxostat vs placebo for 108 wks	467 patients with stage 3 CKD and asymptomatic hyperuricemia	Febuxostat did not mitigate the decline in kidney function among patients with CKD-3 and asymptomatic hyperuricemia (difference of 0.70 with 95% CI, −0.21 to 1.62; *P* = .1)
Beddhu et al^75^ (2016)	Double blinded randomized controlled trial	Febuxostat vs placebo for 24 wks	82 overweight adults with hyperuricemia and type 2 diabetic nephropathy. Percutaneous fat biopsies were performed at 24 wks	No effects were observed regarding subcutaneous adipose tissue oxidative stress and urinary marker levels for fibrosis.

CI, confidence interval; CKD, chronic kidney disease; FEATHER, febuxostat versus placebo randomized controlled trial regarding reduced renal function in patients with hyperuricemia complicated by CKD stage 3; FREED, febuxostat for cerebral and cardiovascular events prevention study; HR, Hazard ratio; SUA, serum uric acid.

Although multiple studies demonstrated the benefits of allopurinol and febuxostat, three randomized controlled trials, 2 involving allopurinol (the preventing early renal function loss study and the controlled trial of slowing of kidney disease progression from the inhibition of xanthine oxidase trial) and one involving febuxostat (the febuxostat versus placebo randomized controlled trial regarding reduced renal function in patients with hyperuricemia complicated by CKD stage 3 study), were negative in terms of nephroprotection.

### Uricosuric drugs

The major uricosuric drugs include probenecid, benzbromarone, and dotinurad. Overall, the number of studies conducted on uricosuric drugs, compared with xanthine oxidase inhibitors, is limited. Benzbromarone is highly effective but is not available in the US because of hepatotoxicity.^54^

A pharmacoepidemiology cohort study conducted by Chou et al^76^ (2018) in Taiwan included 874 patients. The effectiveness of allopurinol, febuxostat, and benzbromarone in reducing the risk of CKD progression to dialysis was assessed. The primary and secondary outcomes were incident end-stage kidney disease and SUA level changes from baseline, respectively. The researchers found that, when compared with allopurinol, benzbromarone therapy was associated with a reduced risk of progression to dialysis (adjusted hazard ratio, 0.5; 95% confidence interval, 0.25-0.99). The authors concluded that, when compared with allopurinol, both febuxostat and benzbromarone were more effective in lowering SUA levels and reducing the risk of progression to dialysis among patients with CKD.^76^

In a single-center, parallel-group, randomized clinical trial conducted by Yu et al^77^ (2018) comparing the safety and efficacy of benzbromarone and febuxostat in hyperuricemic patients with eGFR rates 20-60 mL/min/1.73 m^2^, 66 subjects were enrolled. SUA levels decreased significantly after treatment in both groups. However, after 12 months of treatment, the eGFR rate did not show a significant change in either group. The researchers concluded that both benzbromarone and febuxostat could reduce SUA levels and maintain kidney function in CKD patients with eGFR rates 20-60 mL/min/1.73 m^2^. Interestingly, the authors found that both drugs could increase serum hemoglobin levels and potentially improve anemia.^77^

### Studies examining ULT in patients with asymptomatic hyperuricemia

A network meta-analysis conducted by Sapankaew et al,^78^ included 23 randomized controlled trials of adults with asymptomatic hyperuricemia that compared ULTs (ie, allopurinol, febuxostat, probenecid, benzbromarone, sulfinpyrazone, rasburicase, lesinurad, and topiroxostat) with placebo or no ULT. They found that allopurinol and febuxostat had significantly lower composite renal events than placebo (RR, 0.39; 95% CI, 0.23-0.66 and RR, 0.68; 95% CI, 0.46-0.99, respectively). They also found that both allopurinol and febuxostat treatments resulted in higher eGFR rates than placebo (mean difference [MD], 3.69 mL/min/1.73m^2^; 95% CI: 1.31-6.08 and MD, 2.89 mL/min/1.73m^2^; 95% CI, 0.69-5.09, respectively). Furthermore, they did not find significant differences in adverse events associated with ULTs compared with placebo or no ULT groups. However, it should be noted that they did not include patient populations who are at risk of side effects, such as Korean, Han Chinese, and Thai.^78^

A 2024 network meta-analysis by Chen et al,^79^ which included 12 randomized controlled trials and 4 cohort studies (n = 2,423 patients), was conducted to evaluate the efficacy and safety of febuxostat and allopurinol in people with CKD stage 3-5 and asymptomatic hyperuricemia. Febuxostat was associated with greater improvements in eGFR rates compared with allopurinol (MD, 4.99 mL/min/1.73m^2^; 95% CI, −0.65 to 10.78, low certainty) and placebo (MD, 4.72 mL/min/1.73 m^2^, 95% CI, 0.67-8.82). Major cardiovascular events and adverse events showed no significant differences between febuxostat and allopurinol. The researchers concluded that febuxostat might offer greater improvements in kidney function compared to allopurinol or placebo without compromising safety.^79^

### Uric acid and autosomal dominant polycystic kidney disease

Uric acid and autosomal dominant polycystic kidney disease (ADPKD) are the most common inherited kidney disorders that can lead to end-stage kidney disease. Interestingly, kidney stones are more common in this patient population, with a higher frequency of uric acid stones (40%-60%).^80^ In a retrospective analysis of a prospective observational study at the University of Colorado, which included 680 patients with ADPKD, it was found that high SUA levels were associated with earlier onset of hypertension, larger kidney volume, and increased hazard of end-stage kidney disease in patients with ADPKD.^81^ However, when SUA levels were measured from 671 participants of the halt progression of polycystic kidney disease trial (a randomized, double-blinded, placebo-controlled trial), the investigators did not find that an elevated SUA level was an independent risk factor for disease progression in ADPKD.^82^ The Kidney Disease: Improving Global Outcomes (KDIGO) 2025 guidelines for ADPKD recommend against treating patients with ADPKD pharmacologically for asymptomatic hyperuricemia.^83^

### Guidelines regarding treatment of hyperuricemia

Table 4^32^^,^^84^, ^85^, ^86^, ^87^, ^88^, ^89^, ^90^, ^91^, ^92^ provides a summary of recommendations from various societies/countries regarding the treatment of hyperuricemia.

**Table 4:** Recommendations on Management of Hyperuricemia in Patients With CKD

Society	Recommendation
ESC-2024 hypertension guidelines^84^	Check the SUA level only when investigating hypertension in pregnancy because it is increased in pre-eclampsia
ACC/AHA-2025 hypertension guidelines^85^	Monitor the SUA level in patients taking thiazide diuretics
KDIGO-2024 guidelines^86^	CKD and symptomatic hyperuricemia: - Recommend ULT in CKD after the first episode of gout, especially when there is no avoidable precipitant or SUA level > 9 mg/dL - Xanthine oxidase inhibitors are preferred to uricosuric drugs. Test for HLA-B∗5801 in high-risk patientsSymptomatic treatment of acute gout in CKD: Low-dose colchicine or intra-articular/oral steroids are preferred to NSAIDS CKD and asymptomatic hyperuricemia: Do not suggest ULT to delay CKD progression.
ACR-2020 guidelines^32^	CKD and symptomatic hyperuricemia - The strength of each recommendation was rated as strong or conditional based on evidence, benefits, and risk. - Initiating ULT is conditionally recommended for patients with CKD-3 or more experiencing their first gout flare, SUA level > 9 mg/dL, or urolithiasis. - Allopurinol is preferred over all other ULTs in all patients, including patients with CKD. The guideline strongly recommends starting treatment with low-dose allopurinol (100 mg/d and even lower doses of ≤ 50 mg/d in patients with CKD stage 3 or more) and febuxostat (≤ 40 mg/d) with subsequent dose titration, rather than starting at a higher dose - The guideline conditionally recommends testing for the HLA-B*58:01* allele before starting allopurinol in patients of Southeast Asian descent, such as Han Chinese, Korean, and Thai, and in African American patients. The guideline recommends against universal testing for the HLA-B58:01 allele in other ethnic patient populations.CKD and asymptomatic hyperuricemia Conditionally recommends against initiating ULT in patients with asymptomatic hyperuricemia, including those with CKD
EULAR-2016 guidelines for gout^87^	Symptomatic hyperuricemia and CKD Recommends ULT in patients with patients with a definite diagnosis of gout Asymptomatic hyperuricemia No specific recommendations
CARI-2022 guidelines^88^	Recommend against the use of ULT in people with nondialysis CKD and asymptomatic hyperuricemia (strong recommendation and low certainty of evidence).
Japanese Society of Gout and Nucleic Acid-2019 guidelines^89^	- ULTs are partially recommended (grade B: moderate evidence) for use in hyperuricemia patients with CKD to suppress the deterioration of renal function. - ULTs are not partially recommended for hyperuricemia in patients with hypertension, or to suppress cardiovascular events and heart failure.
IDEA consensus statement-India^90^	- Recommendation is to screen patients with CKD with eGFR rate < 60 mL/min/1.73 m2 for hyperuricemia or patients receiving drugs like thiazide diuretics - Recommend treating asymptomatic hyperuricemia in patients with CKD 3-4. They recommended febuxostat over allopurinol because of the need for dose modification and the increased risk of serious cutaneous reactions with allopurinol - The recommendation for the target SUA level for asymptomatic hyperuricemia is < 6 mg/dL. Avoid SUA level of < 3 mg/dL
Chinese guidelines-2024^91^^,^^92^	Symptomatic hyperuricemia in CKD: - Short-term systemic steroids or intra-articular steroids are preferredAsymptomatic hyperuricemia in CKD: - Recommend treating asymptomatic hyperuricemia in CKD stage ≥ 3 when SUA levels are ≥ 7 mg/dL, aiming for SUA level < 5 mg/dL - Febuxostat is recommended as the first-line treatment and allopurinol as the second line.

ACC, American College of Cardiology; AHA, American Heart Association; ACR, American College of Rheumatology; CARI, Caring for Australians and New Zealanders with kidney impairment; CKD, chronic kidney disease; ESC, European Society of Cardiology; EULAR, European League Against Rheumatism; IDEA, Integrated Diabetes and Endocrine Academy; KDIGO, Kidney Disease Improving Global Outcome; SUA: serum uric acid, ULT, urate lowering therapy.

## Discussion

The incidence of both hyperuricemia and CKD in the general population is increasing. Although there has been some excitement surrounding the use of ULT in asymptomatic hyperuricemia to decrease the progression of CKD, results have been inconsistent, leading many guidelines to not recommend this approach. Currently, the American College of Rheumatology (ACR), KDIGO, and Caring for Australians and New Zealanders with Kidney Impairment guidelines do not recommend using ULT for asymptomatic hyperuricemia in people with CKD. In contrast, Asian guidelines, including Japanese, Indian, and Chinese, have recommended ULT for hyperuricemia in patients with CKD to potentially slow the deterioration of renal function. In fact, in a Japanese cross-sectional study on health insurance claims, ULT was prescribed to 72.4% of patients with asymptomatic hyperuricemia.^93^ Both Indian and Chinese guidelines recommend febuxostat over allopurinol as first-line therapy.

For a provider treating a patient with CKD with symptomatic hyperuricemia, the guidelines are clear and favor treatment even if it is the first episode of gout or urolithiasis. Both KDIGO and ACR guidelines prefer xanthine oxidase inhibitors over uricosurics. HLA-B∗5801 testing is recommended in patient populations at increased risk of adverse events. Regarding the timing of initiation of ULTs, the authors of ACR recommend starting ULT during the gout flare because they consider the benefits of starting therapy to outweigh the risk of flare. Patients are more motivated to take ULT because of symptoms, and treatment decreases the risk of the patient not returning for follow-up. As a rule, ULT should be started at lower doses and titrated up. A “treat to target” strategy to achieve a goal SUA level < 6 mg/dL is recommended by both ACR and European League Against Rheumatism. Both societies recommend concomitant anti-inflammatory prophylaxis ranging from 3-6 months. If a patient cannot achieve the SUA target level of < 6 mg/dL, ACR recommends switching to an alternate xanthine oxidase inhibitor over adding a uricosuric. ACR also recommends continuing the ULT indefinitely rather than stopping if therapy is well tolerated. ACR does not recommend checking urinary uric acid or alkalinizing the urine for patients receiving uricosuric treatment.

For treatment of acute flares of gout in patients with CKD, low-dose colchicine or intra-articular/oral steroids are preferred to nonsteroidal anti-inflammatory drugs per KDIGO guidelines. ACR does not specify which medication is preferred for CKD gout flare. European League Against Rheumatism guidelines specifically mention colchicine, and nonsteroidal anti-inflammatory drugs should be avoided in patients with severe kidney disease.

It is important that in all patients, including those with CKD, simple measures like promoting weight loss and education on limiting alcohol intake and fructose intake, particularly from soft drinks, sugar-sweetened beverages, and high-fructose corn syrup, should be implemented. Concomitant medications should also be reviewed and modified as appropriate; for example, switching hydrochlorothiazide to alternate antihypertensives such as calcium channel blockers or losartan. Losartan is preferred over angiotensin-converting enzyme inhibitors and other angiotensin receptor blockers because of its unique uricosuric properties. If a patient has a history of CVD or new-onset CVD, the ACR conditionally recommends switching from febuxostat to an alternate ULT. “Conditionally recommends” means that the benefits and risks may be more closely balanced and/or have low certainty of evidence. For transplant patients, cyclosporine can cause high SUA levels and should be switched to an alternative agent such as mycophenolate. Use of allopurinol and febuxostat with azathioprine can cause cytopenias and should be avoided.^94^ Tacrolimus has a decreased risk of hyperuricemia when compared with cyclosporine.^95^ Kidney imaging should be reviewed, especially for patients who have had stone analysis showing uric acid stones, since they qualify for ULT.

In conclusion, uric acid possesses both antioxidant and pro-oxidant properties. Reflecting this complexity, studies of the effect of lowering SUA level in slowing the progression of CKD have shown conflicting results. It is unclear if the relationship between SUA levels and these effects is J-shaped or U-shaped, and what target levels would be optimal. More studies are needed to delineate the optimal target SUA levels and to identify patient subgroups who would most benefit from ULT.
